# The dynamics of narrative writing in primary grade children: writing process factors predict story quality

**DOI:** 10.1007/s11145-015-9618-4

**Published:** 2015-12-31

**Authors:** Janne von Koss Torkildsen, Frøydis Morken, Wenche A. Helland, Turid Helland

**Affiliations:** Department of Special Needs Education, University of Oslo, Blindern, Postboks 1140, 0318 Oslo, Norway; University of Bergen, Bergen, Norway; Helse Fonna, Stord, Norway; Statped Vest, Bergen, Norway

**Keywords:** Writing, Development, Process, Key stroke logging, Cognitive abilities, Narratives

## Abstract

In this study of third grade school children, we investigated the association between writing process measures recorded with key stroke logging and the final written product. Moreover, we examined the cognitive predictors of writing process and product measures. Analyses of key strokes showed that while most children spontaneously made local online revisions while writing, few revised previously written text. Children with good reading and spelling abilities made more online revisions than their peers. Two process factors, transcription fluency and online revision activity, contributed to explaining variance in narrative macrostructural quality and story length. As for cognitive predictors, spelling was the only factor that gave a unique contribution to explaining variance in writing process factors. Better spelling was associated with more revisions and faster transcription. The results show that developing writers’ ability to make online revisions in creative writing tasks is related to both the quality of the final written product and to individual literacy skills. More generally, the findings indicate that investigations of the dynamics of the writing process may provide insights into the factors that contribute to creative writing during early stages of literacy.

## Introduction

Writing can be studied from two main perspectives: a product or a process perspective (Berninger, Fuller, & Whitaker, 1996). The product perspective concerns the final written text, for example the content, length or spelling of a written story. The process perspective examines how that text came about. More specifically, studies of the writing process may investigate factors such as the speed of transcription and revisions made to the text. We know a great deal about how children’s written products improve during the school years and the cognitive factors that contribute to the quality of written products (e.g. Berninger et al., 1992; Juel, 1988; Olinghouse, 2008). On the other hand, we know relatively little about how children produce their earliest texts and how their actions during writing relate to the final product. Moreover, it is unclear whether the cognitive factors that children draw on during the writing process differ from the factors which have been found to be important to product measures. The present study set out to investigate how writing process factors relate to product measures and key cognitive skills in Norwegian 8-year-olds.

Writing skills in primary school children are often assessed in word and sentence dictations. However, this gives little information about the individual’s competence on how to use language creatively. The present study focused on another approach to assessment of early writing: the narrative. A narrative is a form of discourse that conveys information about a sequence of (real or imagined) events which are typically embedded into a spatio-temporal context (Peterson & McCabe, 1994; Polanyi, 1982). Since the present study concerns the relation between writing skills and cognitive background factors, it should be noted that previous studies suggest that narrative composition and sentence dictation tasks may draw on different cognitive resources. For example, Bourke, Davies, Sumner, and Green (2014) found that different types of visual processing skills predicted performance in a sentence dictation and a narrative writing task. While sentence dictation tasks provide information of a child’s literacy level, narrative abilities have also come to be regarded as educationally important. There is now a substantial body of literature supporting the link between children’s narrative abilities and their academic performance (for a review, see Boudreau, 2008).

### Process factors in children’s handwriting

According to Berninger et al. (1996), writing involves at least three processes: planning, translating and revising. Planning involves the generation of ideas, organizational strategies and goals. Translating is the transformation of ideas into language (text generation) and written words (transcription). Revising involves an examination of the text already produced and steps to correct or modify it. The present study focuses on the two latter components of the writing process, translation (specifically the transcription process) and revising.

In a study of 6 and 9-year-olds by Sumner, Connelly, and Barnett (2013), transcription fluency, the time it takes a child to transcribe each word in the text, was found to relate to the quality of the written narrative. This study examined the handwriting process in typically developing children and children with dyslexia using a digitizing tablet which recorded the temporal characteristics of writing, including pausing. An additional finding was that for the children with dyslexia, spelling difficulties were significantly related to pausing times and transcription fluency. For typically developing children, however, there was no significant relationship between spelling and the writing process variables pause time, transcription fluency or the product variable narrative quality. In line with this, a recent study by Alves and Limpo (2015), found that neither handwriting nor spelling made a significant contribution to explaining variance in pause length in primary school children between the ages of 7 and 12. For younger children (grades 2–4), however, handwriting and spelling both made a significant contribution to explaining variance in the length of writing bursts, but for older children only handwriting did. The length of writing bursts, in turn, explained significant variance in text quality (narrative or expository) at all grade levels. These two studies demonstrate that the examination of various writing process factors, including transcription fluency and length of writing bursts, can contribute to our understanding of developmental progress in writing.

With regard to revisions, Berninger and Swanson (1994) argue that “Because planning and revision can vary in their scope and when they are done, distinctions need to be drawn between […] local on-line revising and posttranslational local and global revision” (p. 70). Based on their previous empirical studies of children from first grade to junior high school, Berninger and Swanson propose a model of writing development where these types of revisions emerge at different ages. The model suggests that primary school children occasionally make online revisions, but generally do not engage in posttranslational revision. Posttranslational revisions emerge in intermediate grade students at the global text level, and extend to operate at all levels of language (word, sentence, text) in junior high school. Subsequent studies of revision of handwritten texts by children are in line with this pattern (Chanquoy, 2001; Limpo, Alves, & Fidalgo, 2014). However, these studies have generally focused on the effect of different teacher-initiated revisions, and thus less is known about spontaneous revision behavior during the primary school years. Regarding the effect of revisions, previous studies have found that young writers’ revisions have a limited impact on compositional quality (Fitzgerald & Markham, 1987; Limpo et al., 2014). For example, Limpo et al. (2014) found that revision skills did not predict text quality in Grades 4–6, but did have a significant contribution in Grades 7–9. A possible reason why revision does not contribute to text quality at younger ages is that emergent writers tend to focus on transcription and local problems, while older writers attend to meaning and global problems. This focus on transcription and local problems in younger writers may reflect an inability to detect mismatches between their intended text and the text they have written, but may also reflect a problem with executive control, i.e. that children have the necessary competence to diagnose and operate on problems in their writing, but cannot afford to so, because the executive burden so large that a further load would disrupt the composing process (Bereiter & Scardamalia, 1987).

### Key stroke logging of children’s writing

Children are increasingly using computers and mobile devices for writing. In Norway, where the present study was carried out, children are using computers in school from first grade (age six), and are expected to write short narratives and expository texts on the computer from the early primary school years. A meta-analysis of studies comparing writing with computers to writing with paper-and-pencil in children from kindergarten to grade 12, suggested that the writing tool impacts both the way children write and the final written product (Goldberg, Russell, & Cook, 2003). Specifically, the authors found an advantage in favor of computers with regard to both quantity and substantive quality of writing. Moreover, children using computers made more changes to their writing. In addition to the differences in the transcription itself, the ease of reading previously written text, erasing, cutting and pasting text on the computer may place different demands on both the planning and the revision process.

While the advent of computer use in the primary schools may lead to changes in children’s writing, it also opens the possibility of investigating writing as it unfolds in real time through key stroke logging. Key stroke logging programs record the typing behavior of the writer, allowing the researcher to replay and analyze the dynamics of the writing process, such as transcription fluency, pausing and revisions (Wengelin & Strömqvist, 2005). The technique holds promise to expand our understanding of the writing process during typical literacy development, and may also have clinical applications in helping to pinpoint where in the writing process children with language impairments and other disabilities experience the largest difficulties. However, at the present stage, more knowledge about key strokes in typically developing children is necessary before its clinical applications can be fully exploited.

To our knowledge, only a handful of previous studies have investigated the development of writing skills in children through key stroke logging (Asker-Árnason, Wengelin, & Sahlen, 2008; Asker-Árnason et al., 2012; Gnach, Wiesner, Bertschi-Kaufmann, & Perrin, 2007; Morken & Helland, 2013). A main aim in these studies has been to combine the product and process perspectives on writing outlined by Berninger et al. (1996), i.e. to examine the relation between the final text and how that text came about. Asker-Árnason et al. (2008) explored how twenty-seven 8–12 year-old children produced written narratives in online production. The process variables they examined were transition time (writing speed), pauses and transcription fluency (the time it took for a child to produce a word in the final edited text). In addition, they measured three aspects of the final product: the number of words, number of complex clauses and narrative macrostructural quality. The authors found a significant correlation between writing speed and percent complex clauses in the younger age group (8;0–9;11 years). In the older age group (10–12 years), less pause time was associated with higher narrative ability. There were also significant gender differences in the older age group, in both transition time and text flow, with girls being faster. Gnach et al. (2007) reports preliminary results from a pilot project studying primary school children’s writing in a web-based interactive writing environment, but no systematic data analyses are provided in the paper. Morken and Helland (2013) used a structured sentence dictation task to examine differences in writing product (number of errors in spelling, grammar and semantics) and process (transcription fluency and number of revisions) variables in a group of children with dyslexia and controls with typical literacy development at age 11 years. They found that the dyslexia group revised their work equally much as the typical group, and largely in the same way. However, the end products of the dyslexia group were still significantly poorer than those of the typical group. The authors concluded that cognitive factors known to influence reading affect the writing process as well as the final product.

Only two of the above key stroke logging studies investigated the use of writing conventions (spelling, capitalization and punctuation). Gnach et al. (2007) reported that the first to fifth graders who participated in their study generally moved through their text after it was finished and corrected misspelled words and use of capitalization. However, the participants were typically instructed to correct mistakes by their teachers. The study by Morken and Helland (2013), on the other hand, examined the spontaneous use of revisions in school age children, and also included measures of spelling in sentence dictation. Results showed that typically developing children produced fewer spelling errors than children with dyslexia, and that children with dyslexia made even more online revisions attempting to correct spelling than the typically developing group. Although the paper does not report a direct test of the relation between spelling errors and revisions, it suggests that frequent online revisions may be associated with poorer spelling ability in elementary school children.

### Cognitive contributors to writing skills

The studies that have examined the cognitive contributors to written composition in children have typically focused on one or more of the component processes in the ‘Not-So-Simple View of Writing’ by Berninger and Winn (2006). This model specifies four types of cognitive processes involved in writing: (1) text generation (translation of ideas into language representations in memory), (2) transcription (translation of language representations into written words), (3) executive functions (such as supervisory attention, goal setting, planning, reviewing and revising) and (4) working memory, which includes both the storage units for verbal information, the phonological loop for maintaining verbal information in working memory, and executive supports which link verbal working memory with the general executive system.

#### Factors related to text generation: oral language and reading skills

The text generation process is thought to draw critically on both oral language abilities and reading skills. For example, Abbott and Berninger (1993) found that for children in first grade, both verbal reasoning and reading contributed significantly to the quality of narrative composition. In the second and third grades, only reading had a significant contribution, a fact that may have been due to the high covariance between reading and oral language at this age. A number of other studies have demonstrated that oral language skills such as grammatical competence and vocabulary explain significant variance in narrative quality in samples of typically developing children (e.g. Babayiğit & Stainthorp, 2010; Kim, Al Otaiba, Sidler, & Gruelich, 2013; Olinghouse, 2008). While there is mounting evidence of a link between oral language and written narrative quality, the literature is not entirely consistent. In a study of children with specific language impairment, age-matched and language matched controls, Mackie and Dockrell (2004) found no reliable relationship between oral language skills and writing content. Puranik and Al Otaiba (2012), who examined the development of writing skills in kindergarten children, also failed to find a significant contribution of oral language skills (expressive vocabulary and grammar) on the children’s ability to express ideas in writing. They suggested that in kindergarten children, who have had very little writing instruction, may be consumed by the demands of transcription, but that oral language skills may play a greater role in writing in the higher grades.

With regard to the relation between children’s written expression and their reading abilities, empirical findings suggest that reading and writing draw on shared knowledge, yet are separable skills with distinct developmental trajectories (Berninger, Abbott, Abbott, Graham, & Richards, 2002; Fitzgerald & Shanahan, 2000). In a study with 600 unselected children in grades first through sixth, Berninger et al. (2002) found that reading comprehension had a direct, significant influence on compositional quality (narrative and expository) at all grade levels and on compositional fluency (amount of text generated) at the levels 1, 2, 3 and 6. The authors suggested that the ability to understand text may influence both the language representations that children are able to generate and the text generation itself. Children with good reading comprehension may have a greater interest in reading, which in turn may lead to greater interest in composing text and awareness of how authors approach text writing. Consistent with this, another large-scale study of 527 first graders found that reading comprehension was a unique predictor of quality of narrative writing (Kim et al., 2013). Olinghouse (2008), who used word reading ability rather than reading comprehension as a measure of reading, found that reading skill was a unique predictor of narrative quality in third grade school children. However, a few other studies have failed to find a relation between quality or content of narrative writing and measures of reading (Babayiğit & Stainthorp, 2010; Williams & Larkin, 2013). Williams and Larkin (2013) investigated the relationship between a number of reading measures (single word reading, passage reading accuracy, fluency and comprehension) and measures of narrative writing in primary school children. Surprisingly, they found no significant correlations between the quality and content of written narratives and any of the reading measures. However, reading fluency was significantly related to the amount of text children produced. Based on these results, the authors suggest that reading fluency reflects the automaticity of lexical access, and that rapid access to orthographic and semantic information may in turn facilitate children’s translation process. The effect of reading fluency and transcription should thus be especially important when children write under time constraints. In contrast to most previous studies which found a relation between reading and narrative abilities, both Williams and Larkin and Babayiğit and Stainthorp used a series of 6–8 pictures to elicit written narratives. This elicitation mode did not require children to generate story ideas from long term memory and may have reduced the effect of familiarity with written language schemas that are largely acquired through reading comprehension.

Studies specifically aimed at studying the dynamics of the writing process in children have not included independent measures of language or literacy skills, and thus we know little about how language and literacy may influence behavior during the writing process.

#### Transcription factors

Transcription involves transforming the language representations generated by the writer into written symbols. Handwriting/typing and spelling are key components in this process. During composing, low-level transcription and high-level constructive processes must be coordinated in real time. A number of previous studies have found that individual differences in transcription skills during the primary school years predict quality of the written product, perhaps because automatization of transcription may free up working memory capacity that can be devoted to high-level cognitive processes (Berninger, 1999; Graham, Berninger, Abbott, Abbott, & Whitaker, 1997; Puranik & Al Otaiba, 2012).

#### Executive functions: attention

While the executive functions component outlined in early writing models, such as the ‘Simple view of writing’, focus on high-level strategies, the ‘Not so simple view of writing’ also embraces low level executive functions (Berninger & Chanquoy, 2012; Berninger & Winn, 2006). Specifically, the ‘Not so simple view of writing’ incorporates a supervisory attention component, which is involved in selecting what is relevant, inhibiting what is not relevant and switching between mental sets. In the literature on low-level executive functions, a distinction is typically drawn between three types of separable functions: inhibition, shifting and updating (Miyake et al., 2000). Inhibition refers to the ability to deliberately inhibit dominant, automatic, or prepotent responses, thus including selective attention. Shifting concerns switching back and forth between different mental operations, sets, or tasks. The third component, updating, is closely tied to working memory. Updating involves monitoring and coding incoming information for relevance and replacing information that is no longer relevant with newer relevant information.

This system of low level executive functions, which is assumed to underlie higher level executive functions, has only recently begun to receive attention in the writing development literature. However, there is mounting evidence that inhibition, shifting and updating processes play a role in writing development (Altemeier, Abbott, & Berninger, 2008; Altemeier, Jones, Abbott, & Berninger, 2006; Hooper et al., 2011). Inhibition, including selective attention processes, may have a role in suppressing inappropriate lexical items and syntactic structures, as well as keeping relevant items in working memory until they have been transcribed (Altemeier et al., 2008; Drijbooms, Groen, & Verhoeven, 2015). The study by Drijbooms et al., which examined a large range of executive functions in fourth graders, found that inhibition and updating, but not planning, contributed directly to length of written narratives. Moreover, inhibition and updating contributed to both handwriting fluency and spelling, which in turn contributed to syntactic complexity and story content. The finding that executive functions did not contribute directly to syntactic complexity or content was interpreted with regard to the framework of ‘Not-So-Simple View of Writing’ which suggests that for beginning writers the cognitive load of transcription prevents the contribution of executive functions to writing.

Since inhibition, a notion which includes selective attention, has been found to relate to writing development in children, we aimed to assess this facet of executive functions in the present study. There are several ways of assessing attention, but few tests are aimed specifically at language functions (see Miyake et al., 2000, for an overview). However, it has recently been argued that dichotic listening (DL), which is the most frequently used paradigm to assess verbal lateralization and processing in the brain, offers a useful tool to study cognitive control of attention relevant to language and literacy (Hugdahl et al., 2009; Westerhausen, Bless, Passow, Kompus, & Hugdahl, 2015). DL is a non-invasive method which involves dichotic presentations of stimuli, i.e. two different auditory stimuli are presented simultaneously, one to the right ear and one to the left ear. Due to the anatomy of the auditory system, the right ear signal will have a more direct access the speech processing systems in the left hemisphere than the left ear signal, yielding the so-called right ear advantage. By adding instructions (forced trials) asking the participants to report from either the left or the right ear, top-down attentional modulation of the right ear advantage effect is obtained, yielding a measure of cognitive control. Importantly, the DL paradigm involves very short stimulus sequences (typically syllables such as/ba/and/pa/) and thus the working memory load of the task is insignificant. This makes it possible to obtain a measure of attention that is largely independent of working memory.

#### Working memory

While working memory may be regarded as a part of executive functions, specifically linked to the updating function described above (see e.g. Miyake et al., 2000), it is described as a distinct component in the ‘Not-So-Simple View of Writing’. By now a range of studies have found a relationship between verbal working memory and narrative products in children (e.g. Babayiğit & Stainthorp, 2010; Berninger et al., 1992; Bourke & Adams, 2003). Berninger (1999) describe a series of studies showing that working memory contributed significantly to explaining variance in narrative length and quality from primary grade levels through junior high school. The contribution of transcription factors was larger than that of working memory during elementary school years, but gradually diminished with age. Working memory, on the other hand, appeared to have a stable influence across this period. There appears to be little evidence regarding the influence of working memory on writing process factors in children. However, a study by Morken and Helland (2013) found that working memory was associated with transcription fluency in a sentence dictation task, but not with the number of revisions children made.

### The current study

The body of evidence reviewed above show that the majority of studies of early writing has investigated handwritten rather than computer-written samples, and has tended to focus on the final product rather than the process of writing. Against this backdrop, the present study aimed to address the following two main research questions:

1. When children write on a computer, how do writing process variables (transcription fluency, online and posttranslational revisions) relate to the substantive content and use of writing conventions in the final written product?
2. How do the cognitive factors in the ‘Not-So-Simple View of Writing’ (oral language/reading, transcription skills, attention and working memory) relate to writing process and product variables of narratives?

To investigate these questions, we sought to assess the above-mentioned cognitive factors using direct tests and to recruit a sample of developing writers who had equivalent computer-experience.

## Methods

### Participants

Participants were 42 monolingual Norwegian-speaking children (26 males, 16 females; mean age 8;3 years, range 7;9–8;8) attending 3rd grade at a Norwegian elementary school. All children in the sample used the most common of the two official forms of written Norwegian: Norwegian Bokmål. Written information about the study and a consent form was distributed by the school to the parents of all 63 children attending third grade. Consent forms were returned for 43 children, but 1 child was excluded from the data analyses due to having another first language than Norwegian. Analyses of literacy scores collected in a standard school assessment showed that the children who participated in the study did not differ significantly from the children who did not participate in the study with regard to reading comprehension [t(55) = 0.24, *p* = 0.814] or performance in a sentence dictation task [t(55) = −0.35, *p* = 0.730]. One student in the sample was referred for dyslexia assessment at the time of testing, but the results from this child did not deviate substantially from the mean of the group. Moreover, by including children at all literacy levels, we aimed to assess an ecologically valid group of Norwegian-speaking third graders. Parents of participating children had an education level which was close to the national average. Approximately 46 % of participating mothers and 49 % of fathers had a higher education (at least 1 year of college or university studies), compared to 54 % of women and 39 % of men in the relevant age group in the Norwegian population (Statistics Norway, 2013). All the participating children were taught by the same team of teachers and within the same teaching program, and thus all had a similar amount of experience with computers in a school setting. A parental questionnaire which included questions about home computer-use revealed that approximately 60 % of the sample spent 2 h or less on the internet per week, 33 % spent 3–7 h, and 5 % spent more than 8 h per week (2 % did not return the questionnaire). Regarding computer games, approximately 46 % spent less than 2 h a week, 46 % spent 3–7 h, 4 % spent 8 h or more per week.

### Materials and procedure

Approval to conduct the study was granted from the Norwegian Regional Committees for Medical and Health Research Ethics. The test administration took place at school over 2 days and lasted about 2½ h for each child. The test battery was distributed on three separate stations, and the children had a short break between each test station. The authors performed the testing in collaboration with one trained speech-language pathologist, nine Master’s students in speech language pathology, and one philologist who were all trained in the test procedures.

#### Narrative skills

Written narratives were elicited by a sequence of four pictures. The first picture shows a man and a dog who are about to climb the ladder of a slide on a playground. In the second picture, the man goes down the slide. The third picture shows the dog waiting at the top of the slide and the man on the ground stretching his arms out, urging the dog to slide down. The fourth picture is much like the former, but there is now some liquid at the bottom of the slide (instead of sliding down, the dog has peed). The writing task was carried out in a computer lab, where three to six children were tested simultaneously. The task consisted of a picture inspection phase which lasted approximately 2 min and a subsequent writing phase which lasted 10 min. In the picture inspection phase, the participants were first given 1 min to arrange the pictures in an order that made up a story. Subsequently, the children were asked to look closely at the pictures and notice the people, animals and objects in each picture. This was done to prevent them from overlooking important characters or elements in the setting. Following this inspection, they were asked to write a story based on their individually arranged picture sequence. They were explicitly asked to not just describe the pictures, but write a real story. The children were notified when 1 min remained of their writing time. The great majority of the children were able to produce stories that were judged as complete, either by including global concluding statements or by concluding a specific event.

Key stroke logging was used to record the dynamics of the writing process. This was achieved using a specially developed research edition of the software TextPilot (Include, 2012), an internet-based application allowing simultaneous testing of several children at a time. The application was started and stopped centrally by the test administrator, ensuring that all children had the same amount of writing time available. This also secured that children could not start the task before they had reviewed the story line and characters as instructed. To the child, the application appeared as a simple document for entering text. The whole written story was visible to the child while writing, and regular text editing features like moving the cursor via mouse or arrows, and deleting and adding material were available throughout the process.

The product and process measures that were included in the study are described in Table 1. The transcription fluency measure was calculated as seconds per word by dividing writing time (time from first to last keystroke) by the number of words in the final narrative, thus compensating for differences in the number of words each child wrote. We did not include a measure of pause time, as it is difficult to interpret the significance of pauses at this age (Asker-Árnason et al., 2008), and a recent study did not find associations between pause time and measures of the written product (Asker-Árnason et al., 2012). The revision taxonomy was based on that by Berninger and Swanson (1994) described above. Specifically we distinguished between three types of revisions:

**Table 1:** Overview of the writing product and process variables

Variable	Definition
Product measures	
Narrative macrostructural quality	Sum score of seven story characteristics
Story length	Total number of words
Spelling errors %	Percentage of spelling errors in story
Capitalization and punctuation errors %	Percentage of words which required capitalization or period where this was not used
Process measures	
Transcription fluency	Seconds per word
Online revisions	Number of alterations of the rightmost word in the text
Post hoc revisions	Number of alterations of words left of the last word
Text revisions	Number of insertions of words or sentences in previously written text

*Online revisions* any changes made to the word the child is currently working on—i.e. the rightmost word in the text.

*Post-hoc revisions* any changes made to any other single word in the text.

*Text revisions* insertions of new words or sentences in previously written text, i.e. altering the content of the narrative.

Hence, online revisions correspond to Berninger and Swanson’s “local on-line revising”, post hoc revisions correspond to posttranslational local revision, and text revisions correspond to posttranslational global revisions.

The overall quality of narrative macrostructure was scored according to the Narrative Scoring Scheme (NSS) (Heilmann, Miller, Nockerts, & Dunaway, 2010). The NSS assesses seven skill areas which can be combined to provide a single composite score. Three of the skill areas pertain to story grammar: introduction, conflict resolution and conclusion. Two skill areas relate to children’s use of literate language: use of mental state terms and character development. The remaining two skill areas concern children’s use of cohesive ties: referencing and (event) cohesion. Each of the seven skill areas in the NSS is scored on a scale from 1 (minimal/immature) to 5 (proficient). Thus, the lowest possible score was 7 and the highest possible score was 35. Two of the authors performed the NSS scoring, and 24 % of the stories were coded by both authors with an inter-rater reliability (Pearson product moment correlation coefficient) of 0.90. The microstructural process and product measures were derived from the key stroke logging program or counted by hand by two Master’s students. In cases where scores differed between the two students, the story was re-scored by one of the authors.

#### Tests of oral language skills

Receptive vocabulary was assessed with the Norwegian adaptation of the British Picture Vocabulary Scale II (Dunn, Dunn, Whetton, & Berley, 1997; Lyster, Horn, & Rygvold, 2010). The internal consistency reliability for the age group 8;0–8;11 was reported as 0.89. Receptive grammar was assessed by the Norwegian adaptation of Test for Reception of Grammar, which reports an internal consistency reliability of 0.95 (Bishop, 2003; Lyster & Horn, 2009). To measure expressive language (morphology, syntax and semantics), we used the non-normed test Model Sentences from Ringstedmaterialet (Ege, 1984). The procedure for the Model Sentences test is that the administrator presents a model sentence to the first of two thematically related pictures, and the child is asked to construct a similar sentence matching the second picture. A total of 20 sets of sentences of varying complexity are presented. Syntax is scored as either correct or incorrect (one error) while several errors may be scored for morphology and semantics (Helland & Kaasa, 2005). For the present sample, the split half reliability of the Model sentences test was 0.92.

#### Tests of reading and spelling

Carlsten’s reading and dictation test, a widely used screening test developed for Norwegian school children (Carlsten, 2005), was administered by the class teacher as part of a standard school assessment. The story used in the reading task contains 252 words, and has eight cloze tasks in which the reader has to mark the correct alternative among three printed words. The test is not normed, but the manual specifies that a score below 50 words per minute indicates reading problems. The writing test is a sentence dictation containing five sentences with 25 words. More than eight incorrectly spelled words indicate spelling problems. Thus, we obtained two measures of spelling in this study, one from a test performed as part of school assessment and one from the narrative task itself.

#### Nonverbal skills

As a background measure, all participants received the Matrix Analogies Test—Short Form (Naglieri, 1985), a test measuring general nonverbal abilities. The participant is given 25 min to look at 34 incomplete matrices and select the missing portions among several options. The test was administered in groups of three to five children. The internal consistency reliability of the Matrix for ages 8;0–9;11 was reported as 0.89 (Naglieri, 1985).

#### Working memory

Working memory was assessed by the Digit Span task from the Wechsler Intelligence Scale for Children—Third Edition (Wechsler, 1991, 2003). The score used in the present study was the sum of forward and backward digit span. The forward recall condition is seen as a measure of short term memory, while the backward condition is seen as a measure of working memory. However, since preliminary analyses showed that the two conditions correlated equally with the writing scores, the sum raw score was used as a measure of working memory in our analyses. The internal consistency reliability for the age group 8 years was reported as 0.76 (Wechsler, 2003).

#### Selective attention to the right ear in dichotic listening

This test was based on the DL paradigm reported by Hugdahl (2003). The stimuli consists of six CV syllables presented via headphones in pairs, one syllable played in the right-ear (Re) channel and the other syllable played simultaneously in the left-ear (Le) channel. In this way, all possible combinations are presented forming 30 unlike pairs (e.g. ba–ka.) and 6 like pairs (e.g. da–da). On the non-forced (NF) trials, the participant is asked to report the syllable he/she hears the best. In the forced-right (FR) condition, the participant is instructed to focus attention on and report from the right ear. The FR condition is seen as a measure of selective attention, since it acts synergetically with the stimulus-driven NF condition (Hugdahl & Helland, 2013). Thus, the NF condition demands little cognitive control of the verbal stimuli, while the FR condition demands cognitive control as the participant is asked to focus his or her attention. The typical response pattern is higher correct responses reported from stimuli to the right ear versus responses from stimuli to the left ear in both conditions, however with a larger difference in the FR condition compared to the NF condition (Westerhausen et al., 2015). The verbal responses given by the subjects were scored as number of correct responses from the right and left ears, respectively. The scores for each ear were transformed to percentage scores in order to facilitate comparisons with other studies. The FR is not a pure measure of attention, as it is influenced by the child’s degree of hemispheric lateralization for language, and thus the difference between the laterality indices (LI) for the FR and the NF conditions was used as the measure of attention (Passow et al., 2014). The standard LI formula is (Re − Le)/(Re + Le) × 100.

## Results

### Descriptive statistics

A summary of the scores on the cognitive background measures is presented in Table 2. Results on the four cognitive background tests where standard scores are available indicate that this was a typical sample of third graders. With regard to literacy, information from the class teachers on the children’s reading and spelling skills indicated that the distribution of literacy scores was typical for 3rd graders in the fall semester. This observation was supported by a transformation of the Carlsten reading and spelling scores to z-scores using the mean and standard deviations of the raw scores, which showed a normal distribution with a reading mean z-score of 0.00 (SD 0.97), and a mean spelling z-score of −0.02 (SD 1.01) with no statistical difference between the two (*T* test for dependent samples) and a significant correlation between the two scores (r = 0.64, *p* < 0.0001). A transformation of the scores on the expressive language test to z-scores also showed a normal distribution with a mean z-score of 0.0 (SD 1.00). Scores on the dichotic listening measure (forced and non-forced reports from the left and the right ear) were comparable to previously reported scores for this age group (Hugdahl, 2003). As shown in Table 3, there were moderate correlations between the scores on the cognitive background tests, except for the measure of attention in dichotic listening which did not correlate significantly with any of the other variables.

**Table 2:** Mean performance, standard deviations and mean standard score on cognitive tests

Measure	M	SD	Mean standard score (where available)
Nonverbal IQ	16.26	5.51	104
Attention in dichotic listening	15.33	27.04	
Working memory	11.05	2.67	97
Receptive vocabulary	92.02	13.90	99
Receptive grammar (correct blocks)	15.48	2.21	102
Expressive language	20.20	9.80	
Spelling errors in sentence dictation	3.83	2.62	
Text reading (words per minute)	61.13	30.21

**Table 3:** Bivariate correlations between scores on cognitive tests

Measure		2	3	4	5	6	7	8
1. Nonverbal IQ	–	−0.07	0.45**	0.45**	0.55**	0.64**	−0.25	0.45**
2. Attention in dichotic listening	−0.07	–	0.03	−0.08	−0.03	0.03	−0.29	0.21
3. Working memory	0.45**	0.03	–	0.34*	0.37*	0.49**	−0.43**	0.55**
4. Receptive vocabulary	0.45**	−0.08	0.34*	–	0.32*	0.44**	−0.34*	0.32*
5. Receptive grammar	0.55**	−0.03	0.37*	0.32*	–	0.53**	−0.43**	0.49**
6. Expressive language	0.64**	0.03	0.49**	0.44**	0.53**	–	−0.36*	0.49**
7. Spelling errors	−0.25	−0.29	−0.44**	−0.34*	−0.43**	−0.36*	–	−0.61**
8. Text reading (words/minute)	0.45**	0.21	0.55**	0.32*	0.49**	0.49**	−0.61**	–

* *p* < 0.05; ** *p* < 0.01

As Table 4 shows, there was large individual variation in narrative macrostructural quality and story length, and the variability was even higher for the use of writing conventions. On average the participants misspelled more than a fourth of the words they used, but some children wrote virtually error-free stories and others misspelled more than two-thirds of the words. As for the product variables, the children’s transcription fluency varied substantially, but even the fastest child spent more than 5 s per word. One child with a transcription fluency of 50.6 s per word, more than 3 SD from the mean of the group, was classified as an outlier by the outlier labelling rule described in Hoaglin and Iglewicz (1987), and consequently this score was removed from further analyses. All but two children made online revisions to their texts, but only six children made more than 10 revisions. One participant who made 23 online revisions, approximately 4 SD from the mean of the group, was classified as an outlier by the same procedure as above, and the score was removed from further analyses. Only about half of the children made post hoc revisions, and less than a third made text revisions. Since the post hoc and text revisions were so few, and these variables were not normally distributed, they were omitted from the subsequent analyses.

**Table 4:** Descriptive statistics of performance on the writing product and process measures

	Minimum	Maximum	Mean (SD)
Narrative macrostructural quality	13	27	18.54 (3.19)
Story length	12	86	34.41 (15.93)
Spelling errors %	3	69	27.43 (16.37)
Capitalization and punctuation errors %	0	100	68.58 (32.00)
Transcription fluency	5.30	50.60	16.63 (9.38)
Online revisions	0	23	5.76 (4.38)
Post hoc revisions	0	17	2.93 (4.44)
Text revisions	0	5	0.60 (1.25)

### How do writing process factors relate to the final written product?

To identify whether the writing process factors transcription fluency and online revisions that made a significant contribution to explaining variance in macrostructural quality, length or spelling of written narratives, three multiple regression analyses with backward elimination were performed, with macrostructural quality, story length and spelling in narratives as the dependent variables (see Table 5). The *p* value to remove was set at 0.10. The analyses revealed that both transcription fluency and number of online revisions were included in the final models for narrative quality and story length. Children who transcribed faster and made more online revisions, wrote longer stories with higher narrative macrostructural quality. The final model for spelling in narratives included only transcription fluency. Children who transcribed faster produced a lower percentage of spelling errors. However, the number of revisions they made did not significantly predict the spelling in the final story. All variance inflation factors were below 1.1, which suggests no threat of multicollinearity (Hair, Anderson, Tatham, & Black, 1995).

**Table 5:** Summary of multiple regression analyses for writing process measures predicting writing product measures

Outcome measure and predictors	B	SE B	β	t-value	*p* value	R^2^ adjusted
Product: narrative macrostructural quality						
*Full model* = *final model*						0.350
Transcription fluency	−0.24	0.05	−0.58	−4.43	>0.001	
Online revisions	0.29	0.12	0.32	2.45	0.019	
Product: story length						
*Full model* = *final model*						0.451
Transcription fluency	−1.36	0.25	−0.67	−5.56	>0.001	
Online revisions	1.39	0.55	0.30	2.53	0.016	
Product: spelling in narratives						
*Full model*						0.177
Transcription fluency	0.97	0.31	0.46	3.15	0.003	
Online revisions	−0.76	0.69	−0.16	−1.10	0.277	
*Final model*						0.172
Transcription fluency	0.92	0.31	0.44	3.02	0.005	

### How do the writing process and product factors relate to cognitive abilities?

While the narrative product measures were significantly correlated with a number of cognitive abilities, the narrative process measures were only correlated with reading and spelling skills (see Table 6). Children with better reading and spelling abilities made more online revisions than their peers. Moreover, children who were good spellers transcribed faster than their peers. The cognitive measure of selective attention was significantly associated with the ability to use capitalization and punctuation in writing.

**Table 6:** Bivariate correlations between written narrative scores and scores on cognitive tests

Variables	Attention in dichotic listening	Working memory	Receptive vocabulary	Receptive grammar	Expressive language errors	Spelling errors	Text reading
Product measures							
Narrative macrostructural quality	0.19	0.35*	0.40*	0.39*	−0.36*	−0.42**	0.49**
Story length	0.09	0.41**	0.20	0.35*	−0.18	−0.50**	0.45**
Spelling errors in narratives	−0.25	−0.32*	−0.27	−0.35*	0.42**	0.81**	−0.55**
Capitalization and punctuation errors	−0.33*	−0.27	0.19	0.27	−0.27	0.38*	−0.53**
Process measures							
Transcription fluency	0.07	−0.17	0.02	−0.19	0.01	0.38*	−0.13
Online revisions	0.14	0.15	0.28^†^	0.20	−0.13	−0.32*	0.35*

^†^ *p* ≤ 0.09; * *p* < 0.05; ** *p* < 0.01

To identify which of the predictors from the Berninger and Winn (2006) model that made a significant contribution to explaining variance in written narrative products and process measures, a series of multiple linear regression analyses with backward elimination were performed with the cognitive variables oral language (aggregate of receptive vocabulary, receptive grammar and expressive language scores), text reading, working memory, attention and spelling (see Table 7). Although narrative macrostructural quality was correlated with a number of cognitive measures, only oral language skills and spelling were significant predictors in the final model, implying that the other measures did not significantly add to the model’s prediction. The final model for story length included two variables: working memory and spelling. Better working memory and spelling were associated with longer stories. As for prediction of the process variables, only spelling was included in the final models for transcription fluency and online revisions. All variance inflation factors were below 2.1, which suggests no threat of multicollinearity (Hair et al., 1995).

**Table 7:** Summary of multiple regression analyses for cognitive measures predicting writing product and process measures

Outcome measure and predictors	B	SE B	β	t-value	*p* value	R^2^ adjusted
Product: narrative macrostructural quality						
*Full model*						0.289
Oral language	0.39	0.25	0.29	1.56	0.130	
Text reading	0.02	0.02	0.17	0.78	0.442	
Working memory	0.10	0.21	0.09	0.49	0.628	
Attention	0.01	0.02	0.08	0.50	0.618	
Spelling	−0.26	0.25	−0.22	−1.03	0.311	
*Final model*						0.323
Oral language	0.46	0.21	0.35	2.19	0.036	
Spelling	−0.44	0.19	−0.37	−2.33	0.026	
Product: story length						
*Full model*						0.355
Oral language	−0.45	1.20	−0.06	−0.38	0.710	
Text reading	0.09	0.11	0.17	0.84	0.409	
Working memory	1.68	1.03	0.27	1.63	0.115	
Attention	−0.92	0.093	−0.15	−0.99	0.329	
Spelling	−2.92	1.26	−0.46	−2.31	0.028	
*Final model*						0.382
Working memory	2.01	0.90	0.32	2.23	0.033	
Spelling	−2.90	0.93	−0.45	−3.13	0.004	
Process: transcription fluency						
*Full model*						0.082
Oral language	0.55	0.69	0.17	0.79	0.435	
Text reading	−0.03	0.06	−0.13	−0.58	0.570	
Working memory	−0.25	0.59	−0.09	−0.42	0.201	
Attention	0.07	0.06	0.22	1.19	0.244	
Spelling	1.18	0.80	0.34	1.47	0.152	
*Final model*						0.119
Spelling	−1.26	0.54	−0.38	−2.33	0.026	
Process: online revisions						
*Full model*						0.173
Oral language	0.44	0.39	0.22	1.13	0.270	
Text reading	0.30	0.04	0.19	0.82	0.419	
Working memory	−0.04	0.33	−0.02	−0.13	0.899	
Attention	−0.03	0.03	−0.02	−0.11	0.916	
Spelling	−0.50	0.41	−0.27	−1.21	0.236	
*Final model*						0.196
Spelling	−0.085	0.28	−0.47	−3.05	0.005	

## Discussion

This study of 8-year-old children’s narrative writing examined the writing process through key stroke logging and its relation to the final written product. Results showed that while almost all children made local online revisions to their writing, posttranslational revisions were not common. Children with the highest reading and spelling scores made the most revisions. Moreover, the number of online local revisions and transcription fluency predicted narrative macrostructural quality and story length: Children who made many online revisions and transcribed faster, produced better and longer stories. A fast transcription speed also predicted good spelling in the narrative.

Additionally, the present study investigated which cognitive measures (oral language, reading, working memory, attention and spelling) were related to the writing process and product measures. Results showed that only spelling could predict variance in the writing process measures. Spelling was also predictive of the narrative product measures, together with oral language and working memory.

### The relation between process variables and the written product

The finding that a faster transcription process was positively associated with narrative macrostructural quality and other content measures was as expected, and in line with a previous key stroke logging study of primary school children (Asker-Árnason et al., 2008) and a study on children’s handwriting on a digitalizing tablet (Sumner et al., 2013). High transcription fluency also predicted good spelling in the present study. This finding, however, contrasts with the Sumner et al. study which found that transcription fluency was associated with spelling in children with dyslexia, but not in typically developing children. The discrepancy between the two studies may be partly explained by the large differences in spelling abilities between the two samples. The typically developing 9-years-olds in the Sumner et al. study misspelled only 4 % of the words in their written compositions on average, while the corresponding number for the 8-year-olds in the present sample was 27 %. However, a younger control group in the Sumner et al. study (matched on spelling ability with the children with dyslexia) misspelled 37 % of their words, but there was still no significant relation between spelling and transcription fluency in this group. Thus, results for the 8-year-olds in the present study correspond to the pattern of the dyslexics in the Sumner et al. study. The fact that transcription fluency predicted spelling is nevertheless in line with models proposed by Berninger and Swanson (1994) and Berninger (1999) suggesting that spelling skills exert limits on the ability of beginning writers to translate oral language representations to written text.

A surprising finding in the present study was that the number of online revisions made during writing was a unique predictor of macrostructural quality and story length, and that a larger number of online revisions was associated with better products. Consistent with this finding, results from correlational analyses showed that it was children with good spelling and reading skills who made the largest number of online revisions. A previous study found that the number of revisions did not predict narrative quality in grades 4–6 graders, and even in grades 7–9 it was only a certain types of revisions (substantive revisions rather than mechanical revisions) that contributed to writing quality (Limpo et al., 2014). The discrepancy between the results in these two studies may be due to key differences in the tasks used. Limpo et al. (2014) measured revision ability as the number of revisions made to a pre-written text which contained different types of errors. Children were explicitly asked to make revisions to this text, and the number of revisions was compared to the quality of a text written by the student herself. Thus, in the Limpo et al. study the revision process was removed from the burden of generating and transcribing a story. In contrast, the present study examined spontaneous online revisions that were made under the full burden of generating and transcribing a story. Taken together, these results suggest that it may not be the ability to revise per se, but rather the ability to *execute* revisions when they are integrated into the writing process that distinguishes the good primary school writers from the less skilled ones. This finding is in line with the suggestion of Bereiter and Scardamalia (1987) that primary school children have some of the necessary competence to revise, but cannot afford to do so, due to the executive burden. Support for this hypothesis also comes from studies showing that postponing the revision process until a first draft is completed, thus detaching revision from generation and transcription, increases the frequency and depth of revisions in immature writers (Chanquoy, 2001). In our study, it appeared that the children who could afford to engage in online revisions were those who were confident spellers. Automatized spelling may thus reduce the executive load and free resources for detecting and operating on problematic elements in the text (Berninger, 1999).

Our finding that online revisions were related to text quality is also consistent with Bereiter and Scardamalia’s (1987) hypothesis that immature writers employ a knowledge-telling strategy that makes maximal use of oral language competence acquired through conversations, thus resulting in a straight-ahead writing strategy which does not involve revision. With instruction and development, children move towards a knowledge-transformation strategy which involves attention to and deliberate control over the writing process, enabling them to evaluate and modify their texts to obtain a better match with their intended products. In our sample it appeared that awareness of one’s writing process and the accompanying skills for revising text were just emerging after 2 years of schooling, and only those children who had the best reading and spelling skills were able to make use of the revision process to improve their text. Support for the interpretation that revision skills are still emerging during the primary school years, comes from the fact that children in the present study employed mostly only the simplest form of revisions, where they altered the word they were presently working on, and very rarely made changes to previously written text. This finding is in line with Berninger and Swanson (1994) who found post-translational revision to be an emerging skill even in intermediate grade students. Hence, full use of this strategy is not expected at this literacy stage.

A limitation of the current study was the short time allowed for writing (10 min for the narrative task). This resulted in short, and in some cases incomplete, stories. If the children had been given more time to complete their narratives, the scoring of macrostructural quality may have more accurately reflected the narrative competence of the participants. Moreover, the time constraint gave the fast transcribers an undue advantage, and may have contributed to the large influence of transcription fluency on narrative quality. It is also possible that the time constraint was partly responsible for the low number of posttranslational revisions, as many children may not have had the time to read their texts in order identify and correct problems.

### The relation between cognitive abilities and children’s writing skills

The product measures in the present study were related to all the factors in the ‘Not-So-Simple View of Writing’, except the measure of attention in dichotic listening. This finding is in line with previous studies (Berninger, 1999; Berninger et al., 2002; Olinghouse, 2008), and suggests that children largely draw on the same cognitive factors whether they write stories on the computer or by hand. There has been some inconsistency in the literature with regard to the influence of oral language and reading skills on compositional product measures. Specifically, Williams and Larkin (2013) argued that the influence of reading skills on narrative products may be reduced when picture series are used to elicit stories, since this elicitation procedure minimalizes the effect of familiarity with language schemas that are largely acquired through reading. The present study did use a picture elicitation task, but still found that both reading and oral language skills had a significant relation with narrative macrostructural quality. A difference between the present study and that of Williams and Larkin (2013), however, was that the children in the present study were not given a fixed picture sequence. They were required to order the pictures themselves, thus planning their own story line, a task in which may draw on previous story schemas and more generally on individual experience with narratives through reading. As opposed to previous studies which have shown that low-level executive functions predict variance in written products (Drijbooms et al., 2015), our measure of selective attention was not related to the writing measures. The only exception to this was a significant relation between selective attention and use of capitalization and punctuation in narratives. The selective attention task employed in the present study has recently been proposed as a measure of cognitive control (Hugdahl et al., 2009), but there are no previous studies linking it to writing skills. It may be that other facets of attention than the one measured in this task are more closely tied to writing abilities. For example, Drijbooms et al. (2015) found that sustained attention, but not selective attention, was significantly correlated with narrative content. However, this study did find that selective attention was associated with two other measures of writing: text length and handwriting fluency. Moreover, previous studies which have found associations between children’s writing and attention, have used measures of visual attention (Drijbooms et al., 2015; Hooper et al., 2011) which may be more directly related to the activity of writing than the auditory measure used in the current study. Further research with auditory attention tasks will show whether the findings reported here replicate.

As opposed to the writing product measures, the process measures in the present study were correlated only with the cognitive skills spelling and reading, not with oral language, attention, or working memory. Further, the regression analyses showed that spelling was the only unique predictor of transcription fluency and revisions. There is little previous evidence regarding the relation between children’s actions during the writing process and cognitive abilities. However, since several studies have shown that transcription consumes a substantial amount of the young writer’s resources (for an overview, see Berninger, 1999), it follows that automaticity and speed of factors which facilitate transcription may influence both the fluency of this process and the ability to make revisions. Both spelling and reading abilities are key factors that may facilitate transcription. Previous studies have found that spelling has a significant relation with text length and the length of writing bursts in the primary school years (Alves & Limpo, 2015; Graham et al., 1997). Additional evidence comes from the study by Sumner et al. (2013), which showed a significant relation between spelling abilities and the two process variables pause time and transcription fluency in children with dyslexia. The present study adds to these previous findings with English-speaking children by showing the importance of spelling even for a language like Norwegian, which has a semi-transparent orthography (Elley, 1992). With regard to reading abilities, there is some evidence of compromised transcription abilities in individuals with poor reading skills (Connelly, Campbell, MacLean, & Barnes, 2006). It has also been argued that good reading skills contribute to automaticity of lexical access for both semantic and orthographic representations which may in turn enable faster transcription (Williams & Larkin, 2013). Fast lexical and orthographic access may have been especially important in the present study where participants were writing under a severe time constraint.

## Conclusions

Results from the present study indicate that primary school children’s revision behavior during narrative writing predicts the quality of the final written product. Future studies which differentiate functional categories of revisions (corrections of spelling, semantic and stylistic revisions), and which also include different age groups, may further elucidate the role of revision in writing development. Another main conclusion that can be drawn from our study is that spelling appears to be a key factor constraining both the dynamics of writing and the final texts of novice writers. This robust influence of spelling on writing process and product factors is notable since the children in the present study were learning to write in Norwegian, a language with a semi-transparent orthography. Thus, two educational implications of the current study are that spelling instruction should be emphasized even in the early grades of primary school and even in languages with relatively transparent orthographies. Children who do not catch up on their spelling may be severely hindered in their writing process which in turn may compromise their written products.

As opposed to the majority of studies on writing development, the present study investigated writing on a computer rather than by hand. Our findings suggest that the cognitive factors that contribute to quality of handwritten narratives, related to text generation, transcription, and revision (Berninger & Winn, 2006), also contribute to the quality of computer-written narratives. To date, only a handful of studies have used key stroke logging to examine early writing, but the results of the present study indicate that this tool holds promise for expanding our understanding of how primary grade children approach text writing, and how their writing skills develop over time.
